# Sensitivity of major chronic diseases and patients of different ages to the collapse of the healthcare system during the COVID-19 pandemic in China

**DOI:** 10.1097/MD.0000000000040730

**Published:** 2024-11-29

**Authors:** Ayub Qamar, Liu Hui

**Affiliations:** a Department of Laboratory and Quarantine, Dalian Medical University, Dalian, China.

**Keywords:** cancer, cerebrovascular disease, chronic disease, COPD, COVID-19, heart disease

## Abstract

This study evaluates the sensitivity of major chronic diseases to the collapse of the healthcare system for developing prevention and control strategies under normal and emergency conditions. Data for the years 2018, 2019, and 2020 (coronavirus disease 2019 [COVID-19] pandemic) were curated from the National Disease Mortality Surveillance System, Chinese Center for Disease Control and Prevention for diseases such as cancer, heart disease (HD), cerebrovascular disease (CVD), and chronic obstructive pulmonary disease (COPD). The yearly death rate change for 2018, 2019, and 2020 were calculated. Similarly, expected and observed death cases, 95% confidence intervals, and Z-score were calculated for the year 2020 (COVID-19 pandemic). Furthermore, linear regression analysis was performed to analyze a correlation between the median age of various groups and the mortality rate. The observed death cases for cerebrovascular, heart, and other chronic diseases, were more than the expected death cases (430,007 vs 421,317, 369,684 vs 368,957, and 302,974 vs 300,366) as well as an upper limit of 95% confidence interval. The observed death cases for COPD and cancer are less than the expected death cases (127,786 vs 140,524, 450,346 vs 463,961) and lower limit of the 95% confidence interval. The highest Z-score was noted for cerebrovascular disease (105.14). The disease impact of severity was CVD, other chronic diseases, and HD in descending order. The unexpected decline in deaths was found for COPD and cancers with Z-scores (-166.45 and −116.32). The severity of impact was CVD, other chronic diseases, HD, cancer, and COPD in descending order. The COVID-19 pandemic has also resulted in an increase in deaths of the relatively young population as shown by the difference in rate of slop. The healthcare system collapsed due to prevention, control measures and increased burden of COVID-19 patients, affected chronic disease treatment/management and as a consequence variation in death rates occurs in different chronic diseases. A marked increase in mortality was observed in cerebrovascular disease. The unexpected decline in deaths from COPD and cancers, and increase in deaths of the relatively young population suggests that there may be opportunities for improvement in chronic disease management.

## 1. Introduction

Chronic diseases are long-term conditions that require persistent medical attention and management. The management protocol of these diseases is dependent on various factors, including medical treatment, lifestyle changes, and overall health status.^[1–3]^ These diseases include respiratory, heart, cerebrovascular, and cancers. During the coronavirus disease 2019 (COVID-19) pandemic, large numbers of patients with severe acute respiratory syndrome coronavirus 2 (SARS-CoV-2) entered healthcare facilities. This results in a collapse of healthcare resources.^[4–6]^ The highest level of prevention and control measures were taken during the COVID-19 pandemic in mainland China. During COVID-19, hospital facilities i.e. diagnostic tests, and inpatients department services were allocated for COVID-19 patients^[7]^ including the workforce.^[8]^ Regular temperature testing, blood tests, etc were strictly monitored for inpatients.^[9–13]^ Inpatients department elective procedures were widely canceled or postponed.^[14,15]^ Out-patient department services are ceased/decreased to minimize patients’ exposure.^[16,17]^

Cerebrovascular diseases (CVD) cause high mortality and morbidity. Its outcomes intensively depend on the effective delivery of the treatment regime on time.^[18,19]^ The thrombolysis and thrombectomy cases dropped significantly during the pandemic.^[20]^ Heart disease (HD) remains the leading cause of mortality worldwide^[21]^ and ≈70% deaths occur in middle- and low-income countries.^[22,23]^ COVID-19, data revealed pre-hospital delay was increased among the CVD and HD patients while patients’ examination was reduced.^[24,25]^ Heart failure affects approximately 64.3 million people.^[26,27]^ Rapid diagnosis and treatment of acute coronary syndromes are important to avoid further complications.^[28,29]^

Chronic obstructive pulmonary disease (COPD) is a progressive respiratory condition that causes cardiovascular risk, respiratory failure, and lung cancer.^[30]^ Boers reported that the COPD prevalence was 10.6%.^[31]^ The China Pulmonary Health study (2018) reported that the prevalence of COPD was 13.7% in the population aged≥40 years, and an estimated 100 million cases in China.^[32]^ Pleguezuelos et al^[33]^ reported 90% decrease in COPD patients. Pulmonary function test (PFT) plays a key role in the diagnosis of respiratory disorders. The aerosol formation during PFT testing has resulted in a reduction or closure of this test.^[34]^ The definite diagnosis and physicians’ treatment plans markedly depend on this test.^[35–38]^

The estimated number of cases of cancer in 2020 was 19,292,789.^[39]^ Like other specialist clinics, visits to cancer care clinics have been markedly decreased in many countries^[40,41]^ while in the Netherlands and others, national screening programs for various cancers are temporarily halted due to COVID-19.^[42]^ Immediate diagnosis and management for lung and pancreatic cancers, and acute leukemia are necessary while breast, prostate, cervical, and nonmelanoma cancers may not require immediate treatment at their early stage.^[43]^ This decision strongly depends on the expert opinion of the experienced oncologist.

Some chronic diseases are sensitive to immediate medical treatment/management while others do not require such immediate emergency response. However, it is important to understand chronic diseases sensitive to medical conditions/response. The COVID-19 pandemic situation provides an excellent opportunity to understand the above characteristics of chronic disease. It is crucial for prevention and control strategies development during the pandemic. In this study, quantitative and inferential statistical analysis for the mortality of various chronic diseases in 2018, 2019 and 2020 (COVID-19 pandemic) were performed. Data were curated from the National Disease Mortality Surveillance System in Mainland, China. Chronic disease comparison provides a basis to assess the sensitivity of these diseases to the collapse of the healthcare system.

In conclusion, the COVID-19 pandemic has had a devastating impact on healthcare resources and non-communicable disease mortality. However, the unexpected decline in deaths in COPD and cancer patients, an increase in mortality in CVD and HD patients, and an increase in deaths of the relatively young population suggests that there may be opportunities for improvement in chronic disease management. By navigating the COVID-19 crisis, it is crucial to prioritize the health and well-being of all patients.

## 2. Materials and methods

### 2.1. Original data

The data for the years 2018, 2019, and 2020 (COVID-19 pandemic) were obtained from the National Disease Mortality Surveillance System, Chinese Center for Disease Control and Prevention^[44–46]^ and listed Tables 1–3. This data represents more than 73 million subjects. Cancers, ischemic heart disease (HD), cerebrovascular disease (CVD), and chronic obstructive pulmonary disease (COPD) may account for more than 80% of chronic diseases in China.^[47]^ Specific causes of death were classified according to the International Classification of Diseases, Tenth Revised, Clinical Modification Codes. These are: cancer (C00-C97), heart disease (I20-I25), cerebrovascular disease (I60-I69), and chronic obstructive pulmonary disease (J40-J44).^[48]^ The impact of these 4 diseases’ mortality statistics was analyzed to evaluate the sensitivity of the healthcare system collapse during the COVID-19 pandemic in China.

**Table 1:** Age-stratified rates of death from major chronic diseases in the monitored population of China in 2018 (annual deaths per 10^5^ population).

Age groups	Cancer	Heart disease	Cerebrovascular disease	Chronic obstructive pulmonary disease	Other chronic diseases
20~	3.25	0.99	0.95	0.09	4.35
25~	8.40	3.03	2.33	0.16	8.26
30~	16.60	6.65	5.83	0.43	12.62
35~	21.77	8.66	8.20	0.63	13.93
40~	37.36	14.60	15.17	1.43	19.74
45~	72.74	27.01	31.29	3.09	31.70
50~	193.32	67.20	85.16	10.83	71.39
55~	185.88	66.34	84.52	12.88	63.40
60~	412.00	148.84	201.54	44.65	137.41
65~	590.43	254.94	368.79	97.29	219.60
70~	719.75	402.09	591.00	202.46	337.81
75~	846.30	692.88	960.80	369.90	523.13
80~	1144.48	1563.49	1878.24	845.39	1094.72
>85~	1573.48	4581.17	4122.17	2156.71	2992.00
Total*	436,069	341,646	407,001	151,376	274,569

* Total death cases.

**Table 2:** Age-stratified rates of death from major chronic diseases in the monitored population of China in 2019 (annual deaths per 10^5^ population).

Age groups	Cancer	Heart disease	Cerebrovascular disease	Chronic obstructive pulmonary disease	Other chronic diseases
20~	4.75	1.37	1.19	0.08	6.31
25~	7.00	2.53	1.95	0.10	7.01
30~	13.40	5.27	4.58	0.33	10.57
35~	22.59	9.09	8.76	0.54	14.92
40~	40.10	15.94	17.09	1.41	22.06
45~	73.04	27.13	30.79	2.92	32.49
50~	125.99	44.58	55.78	6.48	48.07
55~	213.84	75.62	95.18	12.85	75.20
60~	319.53	116.12	154.92	32.08	106.07
65~	507.25	213.41	301.38	76.53	184.52
70~	751.84	424.97	606.58	189.85	350.72
75~	1043.06	848.17	1159.58	427.35	653.57
80~	1225.45	1638.43	1938.47	818.06	1173.44
>85~	1820.59	5245.11	4611.32	2287.25	3482.06
Total*	449,799	355,039	414,097	145,849	287,178

* Total death cases.

**Table 3:** Age-stratified rates of death from major chronic diseases in the monitored population of China in 2020 (annual deaths per 10^5^ population).

Age groups	Cancer	Heart disease	Cerebrovascular disease	Chronic obstructive pulmonary disease	Other chronic diseases
20~	4.82	1.69	1.26	0.07	6.51
25~	6.63	2.63	1.89	0.13	7.09
30~	12.61	5.57	4.45	0.26	10.74
35~	21.37	9.74	9.16	0.44	15.97
40~	39.07	16.37	17.66	1.03	22.47
45~	67.73	26.56	30.55	2.43	33.81
50~	118.29	44.36	55.82	5.60	50.80
55~	197.24	70.42	92.01	11.11	75.48
60~	309.63	114.53	154.51	26.21	110.75
65~	485.82	205.24	295.11	62.73	186.16
70~	720.75	401.59	592.21	155.66	351.83
75~	1005.52	818.83	1123.08	351.61	645.15
80~	1259.72	1728.11	2037.84	723.25	1249.71
>85~	1498.15	4613.51	3966.24	1647.83	3009.25
Total*	450,346	369,684	430,007	127,786	302,974

* Total death cases.

### 2.2. Analysis of the death case

The rate of change for the death of CVD, HD, COPD, and cancer diseases were analyzed for the years 2018, 2019, and 2020. The formulae for the change rate and expected death cases were as follows:


$$\begin{array}{c} \text{Change rate}\;2019=[\left(\text{death cases }2019\right)\\ \;\;\;\;\;\;\;\;\;\;\;\;\;\;\;\;\;\;\;\;\;\;\;\;\;\;\;\;\;\;\;\;\;\;\;\;\;\;-\left(\text{death cases }2018\right)/\left(\text{death cases in }2018\right)]\\ \;\;\;\;\;\;\;\times 100\% \end{array}$$

The change rate in 2019 is an expected change rate in 2020; therefore, the expected death cases in 2020 can be calculated as follows:


$$\begin{array}{c} \text{Expected death cases }2020=\left(\text{death cases}\;2019\right)\\ \;\;\;\;\;\;\;\;\;\;\;\;\;\;\;\;\;\;\;\;\;\;\;\;\times (1+\text{Change rate}\;2019/100) \end{array}$$

For each specific disease the *Z* score is calculated as follows:


$$\begin{array}{c} Z\text{ score}=(\text{Observed death cases }2020\\ \;\;\;\;\;\;\;\;\;\;\;\;\;\;\;\;\;-\text{Expected death cases }2020)/\text{SD} \end{array}$$

where SD represents the standard deviation. An increase in *Z* score shows increased observed death cases as compared with expected death cases.

### 2.3. Population age-wise threat analysis

A linear regression was performed to determine a linear relationship between the median age of an age group intervals and mortality (log mortality) for cancer, HD, CVD, COPD, and other chronic diseases. In this regression model, morality is a dependent variable (*Y*) and the median age of the group interval is an independent variable (*X*). The slope for each disease was calculated. The lower slop provides greater weight to the threat of a disease for younger age groups. The difference in rate for slop can be calculated as follows:


$$\begin{array}{l} Dif\;ference\;in\;rate\left(\%\right)=Change\;rate\;2020-Change\;rate\;2019 \end{array}$$

The lower the difference of rate for slop, the greater the threat to the younger population.

The regression equation and 95% confidence interval (CI) were analyzed by the SPSS statistical analysis software (SPSS 17.0, IBM, Armonk). *P* values <.05 were considered significant.

## 3. Results

**Figure 1.**
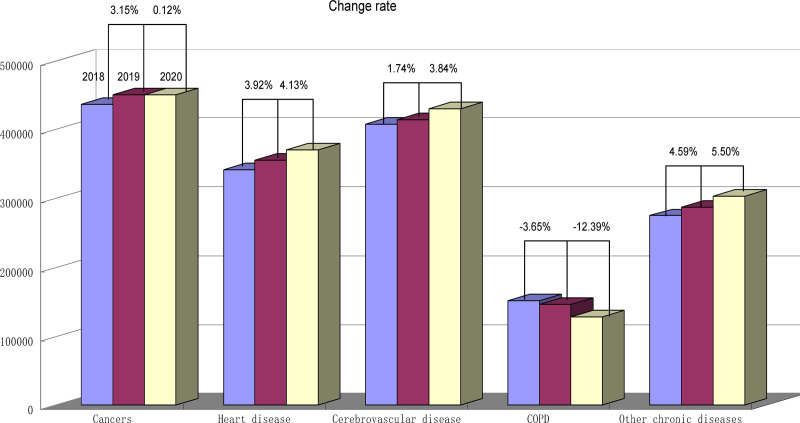
Change rate of mortality for the stipulated chronic diseases.

COVID-19 pandemic has led a devastating impact on the health sector beyond the SARS-CoV-2 patients. Patients including SARS-CoV-2 were extremely affected. Age stratified rates of death from major chronic diseases mortality are mentioned in Tables 1–3. The Change rates for the stipulated chronic diseases are summarized in Figure 1. The change rate for CVD for the years 2018 and 2019 is 1.74% while between the years 2019 and 2020 is increased (3.84%). Similarly, an increasing trend is observed in the HD death change rate for the year 2018 vs 2019 and 2019 vs 2020 (3.92% and 4.13%, respectively). A 3.5% increase in the death rate is noted in cancer from the year 2018 to 2019 (3.15%) while a slight increase between the year 2019 and 2020 (0.12%). In COPD, there is a decreasing trend in the death change rate from the years 2018 to 2020 (−3.65% and −12.39%, respectively) while a marked decrease in the death change rate in 2020 as compared to 2019. Other chronic diseases also show an increasing trend from the years 2018 to 2020 (4.59% and 5.50%).

Expected and observed death cases in 2020 are listed in Table 4. Observed death cases of CVD (430,007 vs 421,317), HD (369,684 vs 368,957), and other chronic diseases (302,974 vs 300,366) are more than the expected death cases and > 95% CI, as shown in Table 4. The observed death cases for COPD (127,786 vs 140,524) and cancer (450,346 vs 463,961) are less than expected death cases and < 95% CI. The *Z* score for CVD is markedly increased (105.14) and vice versa for COPD (−166.45). The *Z* score for HD is 6.41. The cancer *Z* core shows a negative trend that is, −116.32.

**Table 4:** Expected and observed death cases in 2020 (COVID-19 pandemic).

Diseases	Expected death cases	95% confidence interval	Observed death cases	*Z* score
Cancer	463,961	464,191 ~ 463,732	450,346	−116.32
Heart disease	368,957	369,179 ~ 368,735	369,684	6.41
Cerebrovascular disease	421,317	421,479 ~ 421,155	430,007	105.14
COPD	140,524	140,674 ~ 140,374	127,786	−166.45
Other chronic diseases	300,366	300,580 ~ 300,152	302,974	23.88

COPD = chronic obstructive pulmonary disease.

**Figure 2.**
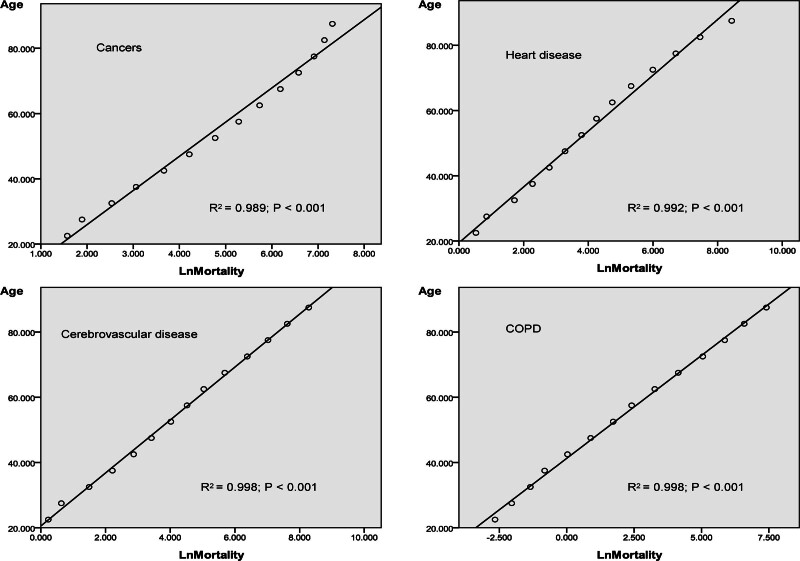
A linear quantitative relation was established using the logarithm of mortality (LnMortality) as *Y* and median age of age groups as *X*.

A line regression equation for cancer, HD, CVD, and COPD was analyzed between the median age of each age group as *X* and the logarithm of mortality (log mortality) as *Y* (Fig. 2). The *R*^2^ values for cancer, HD, CVD, and COPD are 0.989, 0.992, 0.998, and 0.998 respectively. The *P* value for each of these diseases is < .001. The index of change rate and difference of rate for slop are summarized in Table 5.

**Table 5:** Slop of expected rate, observed rate and difference of rate.

Diseases	Slop			Change rate (%)		Difference of rate‡ (%)
	2018	2019	2020	Expected*	Observed†	
Cancer	0.097	0.096	0.095	−1.008	−0.554	0.454
Heart disease	0.118	0.119	0.117	0.607	−1.835	−2.443
Cerebrovascular disease	0.124	0.124	0.123	0.510	−0.924	−1.434
COPD	0.157	0.161	0.159	2.708	−1.618	−4.325
Other chronic diseases	0.095	0.096	0.094	1.351	−1.761	−3.112

COPD = chronic obstructive pulmonary disease.

* Expected rate = (2019–2018)/2018.

† Observed rate = (2020–2019)/2019.

‡ Different rate = observed rate − expected rate.

A lower difference in the rate for slop indicated that the disease could relatively be more threat to the younger population.^[49,50]^ In ascending order, differences in rates for slop were COPD, other chronic diseases, HD, CVD and cancers.

## 4. Discussion

The COVID-19 pandemic has caused an unprecedented strain on healthcare systems worldwide.^[51]^ With the influx of COVID-19 patients into healthcare facilities, resources have been stretched to their limits, resulting in a collapse of healthcare services.^[52]^ However, the impact of the pandemic extends beyond COVID-19 patients. The pandemic has also led to either increase or decrease in various chronic disease-related deaths. Among these diseases, some are markedly affected than others. In general, chronic diseases are long-term conditions that require ongoing medical attention and management.

An increase in the observed cerebrovascular death rate as compared with the expected death rate was noted during the COVID-19 pandemic in the present study (430,007 vs 421,317). The *Z* score is the highest for cerebrovascular disease among specified types of diseases (105.14). Similarly, Sharma et al,^[53]^ reported excess cerebrovascular mortality above the expected mortality level while noted that a 4.3% increase in stroke deaths is due to a 10% increase in time spent at home. Inpatients and out-of-hospital patients fatality rate was higher during lockdown as compared to 2017 to 2019 while 60% of patients were under the age of 65 years.^[54]^ Observed heart disease mortality (369,684) was also more than the expected deaths (368,957) which is more than the upper limit of 95% CI (369,179). This result is consistent with Maung and Marques-Vidal, 2023.^[55]^ The observed death cases for COPD and cancer are less than the expected death cases (127,786 vs 140,524 and 450,346 vs 463,961) and <95% CI. COPD consistent results were reported by others.^[56,57]^ Barbiellini et al^[58]^ reported a decline in neoplasm mortality in the second wave of the COVID-19 pandemic. The *Z* score for heart diseases is 6.41 while for COPD and cancer is −166.45 and −116.32 respectively. This shows that cerebrovascular disease has the highest death rate among these 4 diseases. A descending order with severity of impact exists and is as: cerebrovascular disease, other chronic diseases, heart diseases, cancers and COPD.

The COVID-19 pandemic situation results in an increased risk of patients with acute myocardial infarction and stroke not receiving appropriate acute therapy.^[59]^ Studies revealed that decrease in hospital admissions and thrombolysis/thrombectomy cases.^[20]^ Various studies reported a prolonged time from stroke onset to hospital arrival.^[60]^ Some researchers reported that prehospital delays and decreased admission for stroke care are due to fears among patients and their families regarding SARS-CoV-2 virus infection.^[61]^

However, in China, the highest level of prevention and control measures was taken during the COVID-19 pandemic^[62]^; excess cerebrovascular mortality in China could suggest that cerebrovascular diseases are the most sensitive to the highest level of prevention and control measures in the COVID-19 pandemic.

Unexpectedly, deaths from COPD and cancer decrease during the COVID-19 pandemic. Results suggest that the lockdown was associated with a reduction in COPD exacerbations and an improvement in symptoms.^[63,64]^ The reasons for this unexpected decline in deaths are not yet fully understood. These factors could have led to the underestimation of the true incidence of some diseases. However, death cases were strictly monitored. Therefore, we believe that the data from the National Disease Mortality Surveillance System would be reliable.

One possible explanation is that other respiratory infections were also reduced with strict control during the COVID-19 pandemic in China^[62]^ and patients with COPD may have been more cautious during the pandemic, avoiding exposure to respiratory viruses. Ko et al^[65]^ reported that as there was a decrease in air pollutant levels and influenza rate resulted in a decrease in hospitalizations of COPD and non-COVID pneumonia.

The pandemic has also led to an increase in deaths of the elderly population^[66,67]^; however, the results at present are inconsistent with the previous studies. The pandemic has led to an increase in deaths of the relatively young population except deaths from cancer (Table 5). It is indicated that the younger population are also more sensitive to medical conditions. This result is consistent with Lesaine et al^[68]^ Rudilosso et al,^[69]^ also observed that younger age patients were found in stroke hospital admissions during the pandemic.

In 2020, the total number of deaths from COVID-19 reported in China, was only 4634.^[62]^ Therefore, the collapse of healthcare resources during the COVID-19 pandemic has the potential to affect patients suffering from other diseases, constituting an indirect hazard that sometimes far exceeds the direct hazard of COVID-19 infection in humans.

One of the major limitations of this study is that the reasons for the different sensitivity of major chronic diseases and patients of different ages to the collapse of the healthcare system have not been precisely known. However, understanding the above characteristics of chronic diseases is crucial for developing prevention and control strategies. Prevention and control strategies for chronic diseases should take into account the sensitivity of the disease and population to medical conditions and the general health level. For diseases that are sensitive to medical conditions, prevention and control strategies should focus on improving the level of medical treatment. For diseases that are not sensitive to medical conditions, prevention and control strategies should focus on improving the general health level.

## Author contributions

**Conceptualization:** Liu Hui.

**Data curation:** Liu Hui.

**Writing – original draft:** Ayub Qamar.

**Writing – review & editing:** Liu Hui.
